# Return to Sport and Work Following Distal Femoral Varus Osteotomy: A Systematic Review

**DOI:** 10.1177/15563316211051295

**Published:** 2021-10-27

**Authors:** Hassaan Abdel Khalik, Darius L. Lameire, Luc Rubinger, Seper Ekhtiari, Vickas Khanna, Olufemi R. Ayeni

**Affiliations:** 1 Michael G. DeGroote School of Medicine, McMaster University, Hamilton, ON, Canada; 2 Division of Orthopaedic Surgery, McMaster University, Hamilton, ON, Canada

**Keywords:** distal femoral varus osteotomy, distal femur osteotomy, valgus knee, return to work, return to sport

## Abstract

*Background:* Distal femoral varus osteotomy (DFVO) is an effective surgical intervention for the management of symptomatic valgus malalignment of the knee. Because it preserves the native knee joint and its ligamentous stability, DFVO is preferred to total knee arthroplasty (TKA) in the young, active population. *Purpose:* We sought to assess return to work (RTW) and return to sport (RTS) rates following DFVO for valgus malalignment of the knee. *Methods:* For this systematic review, we searched EMBASE, MEDLINE, and Web of Science from inception through December 31, 2020. English language studies of all levels of evidence explicitly reporting on RTS and RTW rates following DFVO for valgus malalignment of the knee were eligible for inclusion. *Results:* Seven studies and 127 patients were included in our analysis. Mean age was 32.4 ± 8.8 years with men comprising 46.7% ± 22.3% of study populations. The mean RTS rate was 87.2% ± 10.7%, with a return to preoperative activity levels rate of 65.4% ± 26.8%. The mean RTW rate was 81.8% ± 23.3%, with a return to preoperative activity levels of 72.8% ± 18.1%. The mean reoperation rate was 35.6% ± 18.8% within a mean follow-up period of 5.5 ± 1.9 years. *Conclusions:* This systematic review of low-level studies found DFVO to be a safe and effective procedure for the management of genu valgum in young, active populations, with most patients returning to sport and/or work, although not all at their preoperative activity levels. A paucity of data surrounds RTS and RTW rates following DFVO. Future studies should explicitly report both return to activity rates and whether patients returned to their preoperative activity levels.

## Introduction

In the United States, the prevalence of osteoarthritis (OA) has nearly doubled over several decades and now affects more than 32.5 million adults [38,53]. For those with OA globally, knee OA has accounted for over 80% of the total OA burden in years lived with a disability, with a global prevalence rate exceeding 3.5% [37,56]. The prevalence of knee OA is projected to increase, in part due to an aging population and increasing body mass index [14,57]. Knee OA with valgus alignment beyond the physiological 5° to 8° can lead to altered load bearing within the knee joint, which results in increased loading of the lateral compartment [7,17]. Over time, this can progress to chondral and meniscal damage, worsening valgus deformity, and, ultimately, worsening of OA [15,17]. These changes are often accompanied by increased pain, stiffness, and functional impairment [45].

The definitive treatment for multicompartmental OA secondary to genu valgum is a total knee arthroplasty (TKA) [45,61]. Due to the irreversible bone-removing nature of this procedure and its potential to require revision surgery in younger patients, TKA is typically recommended for patients over the age of 60 or in patients for whom other treatments are contraindicated or have failed [45]. For younger and more active patients, other surgical options include partial knee replacements, distal femoral varus osteotomy (DFVO), and high tibial osteotomy (HTO). Some of these procedures, such as DFVO and HTO, preserve the native joint and may delay the need for a TKA [24,44,45]. The HTO is commonly performed for varus deformities or in mild valgus deformities to minimize the risk of future joint-line obliquity [26,44]. However, in cases of idiopathic genu valgum, a medial closing-wedge HTO may be unable to restore a parallel joint line while also resulting in reduced bone stock and possible patella alta [60]. Similarly, for greater degrees of correction, a lateral opening-wedge HTO has a risk of peroneal nerve injury and patella baja [54]. In such cases of genu valgum, and particularly at higher degrees of angulation (greater than 12°), DFVO is preferred [18].

The DFVO is generally indicated for young (<65 years old), active patients suffering from congenital malalignment or isolated lateral arthritis due to idiopathic or posttraumatic deformity [32,40,46]. Relative contraindications include nonunion risk factors (ie, smoking, diabetes, obesity, inflammatory arthropathies, and excessive alcohol use), severe patellofemoral OA, instability due to ligamentous injury, lateral compartment bone loss, previous septic arthritis of the knee, and valgus deformities greater than 20° (although the operation may be a part of a multistep treatment plan) [10,40,46,50]. The DFVO is commonly performed in association with other soft-tissue procedures, including ligament reconstruction, meniscal transplant, and cartilage repair [12,39,50,55]. The goal of the DFVO for genu valgum is to correct the mechanical axis of the knee and offload the lateral compartment, thereby slowing degeneration, decreasing pain, protecting any chondral or meniscal procedures, and delaying TKA [45].

The DFVO is indicated for a population in which return to sport (RTS) and return to work (RTW) have a profound impact on economic implications and quality of life. Previous systematic reviews have assessed the outcomes of DFVO for genu valgum but none to-date have focused on RTW or RTS [7,60]. An increasing focus on value-based outcomes has focused consideration on RTS and RTW in the evaluation of surgical procedures, especially in the younger, more active population [6,8,13,47,51]. Therefore, the primary goal of this study was to evaluate rates and timelines of RTS and RTW following DFVO for valgus malalignment.

## Methods

This systematic review was performed according to the guidelines set out by the Cochrane handbook [19] and is reported according to the preferred reporting items for systematic reviews and meta-analyses (PRISMA) [35].

A systematic search of 3 electronic databases—the Excerpta Medica Database (EMBASE), the Medical Literature Analysis and Retrieval System Online (MEDLINE), and Web of Science—was performed by 2 reviewers for literature related to DFVO for the valgus knee. The search was dated from the inception of each database through December 31, 2020. The search terms included “distal femoral osteotomy,” “varus osteotomy,” and “valgus knee.” The search strategies used for each database can be found in the appendix. The inclusion criteria for this review were (1) all types of varus-producing distal femoral osteotomies; (2) rates reported explicitly for RTS, RTW, or activity; (3) patients diagnosed with lateral compartment pathology (ie, OA or meniscal damage); (4) levels of evidence I to IV; (5) clinical and/or functional outcomes reported; (6) a population that was skeletally mature; (7) human studies; and (8) studies published in English. Exclusion criteria consisted of (1) derotation or gradual osteotomies, (2) diagnosis of patellofemoral instability or pediatric deformities, (3) cadaveric studies, (4) biomechanical studies, (5) no follow-up/outcomes data reported, and (6) review papers. If papers included the same patient population, only the most recent paper was included for analysis, unless there were mutually exclusive patient selection criteria and/or reported outcomes.

Two authors (H.A.K. and D.L.L.) independently screened the titles and abstracts of the identified studies using the inclusion and exclusion criteria described above. To prevent premature exclusion, disagreements were advanced to the full-text review stage. A third author resolved any full-text disagreement (L.R.). After each screening stage, a Kappa (κ) score was calculated to determine the level agreement between reviewers. In reference to a previous study, the categorization of κ scores was defined a priori as follows: 1.00 > κ ≥ 0.80 indicates almost perfect agreement, 0.80 > κ ≥ 0.60 indicates substantial agreement, 0.60 > κ ≥ 0.40 indicates moderate agreement, 0.40 > κ ≥ 0.20 indicates fair agreement, 0.20 > κ ≥ 0.00 indicates slight agreement, and a κ score = 0 indicates no agreement [30].

All of the studies in this systematic review used nonrandomized methodology. Two authors (H.A.K. and D.L.L.) independently assessed the quality of each study using the methodological index for nonrandomized studies (MINORS) [52]. The MINORS questionnaire consists of 12 items for comparative studies, and 8 additional items for noncomparative studies. Each item is scored as 2 if reported and adequate, 1 if reported but inadequate, and 0 if not reported. Comparative studies have a maximum score of 24, and noncomparative studies have a maximum score of 16.

Two reviewers were involved in data abstraction (H.A.K. and D.L.L.). Each reviewer abstracted data from half of the studies; the accuracy of their data was then reviewed by the other reviewer. The data were abstracted into a Google Sheets spreadsheet designed a priori. The following data were recorded: study characteristics (authors, study design, publication year, etc), number of patients, patient demographics (age, sex, etc), follow-up length, details of the surgery performed (opening wedge, closing wedge, etc), subjective outcome measures (Lysholm score, Tegner score, Visual Analog Scale [VAS], Numeric Rating Scale [NRS]), and RTS, RTW, or activity rates. The level of evidence for each study was determined based on guidelines from the American Academy of Orthopaedic Surgeons (AAOS) Evidence-Based Practice Committee [59].

The primary outcome of this study was evaluation of the RTS or RTW for patients undergoing DFVO for a valgus knee. We reported both RTS and RTW rates, whether or not the preoperative activity level was recorded. In addition, when applicable, RTS and RTW rates were reported as return to the preoperative activity level or better. The time to postoperative RTS and RTW was also evaluated.

The secondary outcomes of this study were pain (based on validated outcome measures), radiographic alignment of the osteotomies, and concomitant procedure and reoperation rates. The following pain-related outcomes were abstracted: (1) single assessment numeric evaluation (SANE) score, (2) VAS/NRS pain score, (3) Lysholm score, and (4) International Knee Documentation Committee (IKDC) subjective score. The SANE score asks patients to rate their current functional ability between 0 and 100; 100 represents normal function before injury [3]. The VAS/NRS pain score is a widely used unidimensional scale from 0 to 100 mm or 0 to 10, in which higher scores correspond to greater levels of pain [23,34]. The Lysholm score assesses outcomes of knee ligament surgery and knee instability on a scale from 0 to 10; the maximum score of 100 represents no symptoms or disability [29,33]. The IKDC subjective score is a validated patient-outcomes questionnaire developed to detect the improvement or deterioration of knee function and symptoms after knee impairment on a scale from 0 to 100; 100 represents normal function [9,25]. Concomitant procedures were defined as any therapeutic procedure that took place alongside the index DFVO. Reoperations were calculated based on the number of secondary operations each knee underwent following the index DFVO, irrespective of the number of the procedures that were completed during each reoperation.

### Statistical Analysis

Descriptive statistics were derived using R (RStudio, Boston, Massachusetts). These included weighted means and standard deviations (SDs), as well as 95% confidence intervals (CIs). Depending on the outcome, either the number of patients or the number of knees in each study were used as the frequency weights.

## Results

**Fig. 1.**
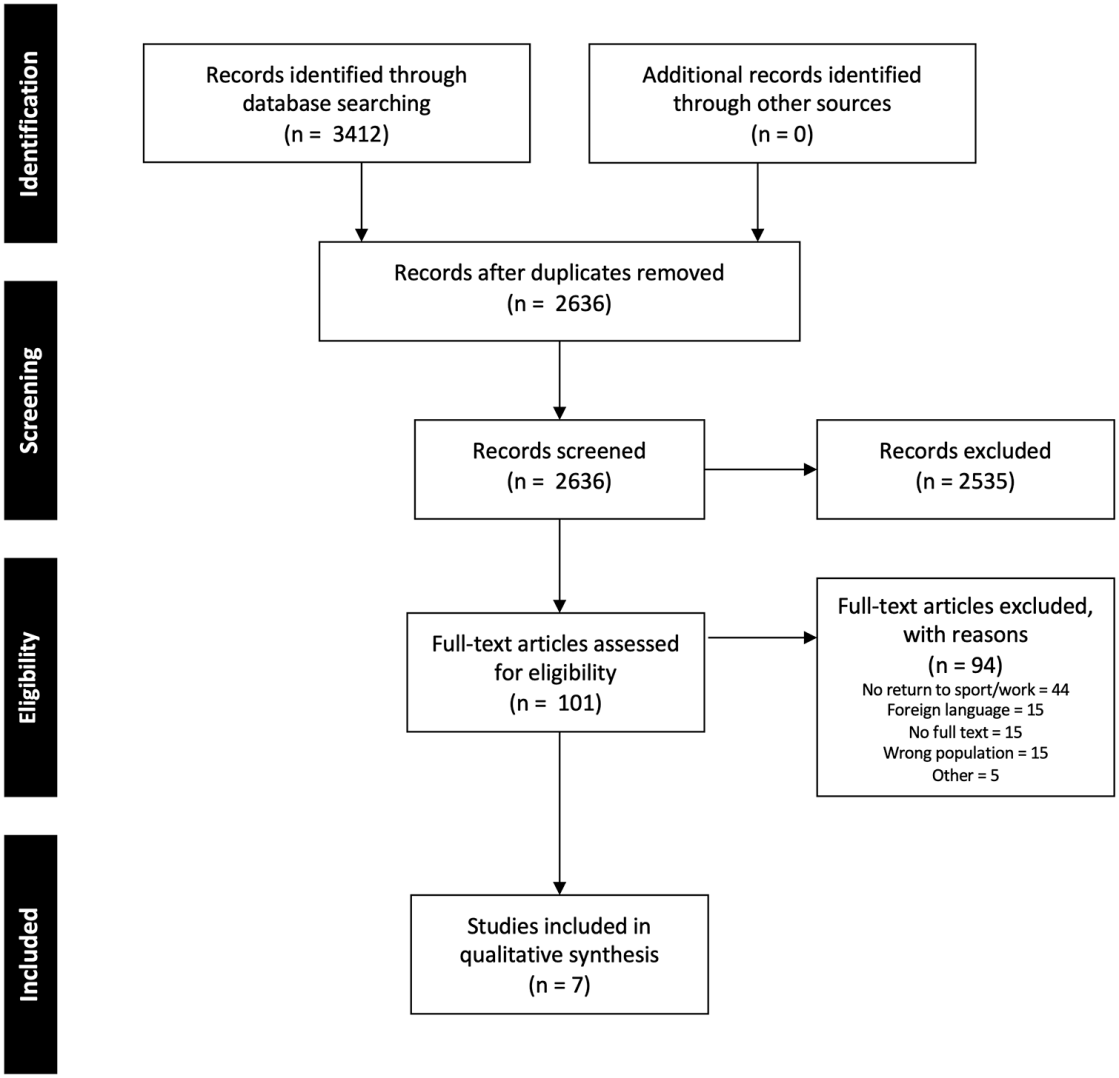
Preferred reporting items for systematic reviews and meta-analyses flow diagram.

There were 3412 studies identified in our search, with 2636 remaining after duplicates were removed. After applying inclusion and exclusion criteria, we identified 7 studies that were eligible for this review (Fig. 1) [1,2,11,41–43,55].

Title and abstract screening had substantial inter-reviewer agreement as the κ score was 0.71 (95% CI, 0.63–0.79), and after the full-text screening, there was almost perfect agreement (0.86; 95% CI, 0.68–1.00). All 7 studies were level IV noncomparative case series. The average MINORS scores for included studies was 11.9 ± 1.6, or moderate quality (Table 1).

**Table 1.** Study and patient characteristics.

Study	Study design	LOE	RTS or RTW study?	Procedure (%)	No. of patients, n (knees)	% male	Age, years (±SD, range)	BMI, kg/m^2^ (±SD, range)	Follow-up, years (±SD, range)	MINORS
Agarwalla et al [1]	Case Series	IV	RTS	Lateral open wedge: 100	17 (17)	52.9	32.1 (10.1, 13.5–46.4)	30.5 (9.1, NR)	7.3 (4.4, 2–13.8)	12.5
Baron et al [2]	Case Series	IV	RTS	Lateral open wedge: 100	3 (3)	33.3	19 (2, 17–21)	NR	3 (1.7, 1.0–4.0)	8.5
de Carvalho et al [11]	Case Series	IV	RTS & RTW	Wedgeless V-shaped: 100	26 (26)	30.8	48.6 (10.6, 21–65)	NR	4 (2.0, 1.7–9.5)	11
Puzzitiello et al [41]	Case Series	IV	RTS	Lateral open wedge: 100	17 (17)	23.5	23 (6.3, 16.9–36.2)	26.4 (4.2, NR)	7.5 (4, 2.2–13.3)	12.5
Puzzitiello et al [42]	Case Series	IV	RTW	Lateral open wedge: 100	32 (32)	34.4	30.8 (8.8, 17.2–46.5)	28.9 (7.2, NR)	7.1 (4.1, 2.2–13.3)	12.5
Rensing et al [43]	Case Series	IV	RTW	Lateral open wedge: 90.9 Medial closing wedge: 9.1	19 (22)	89.5	30 (9.0, 19–50)	NR	3.2 (NR, 0.5–6)	13
Voleti et al [55]	Case Series	IV	RTS	Lateral open wedge: 53.8 Medial closing wedge: 46.2	13 (13)	61.5	24 (NR, 23–31)	27.4 (NR, 23–31)	3.6 (NR, 2–6.2)	13

*LOE* level of evidence, *RTS* return to sport, *RTW* return to work, *BMI* body mass index; *MINORS* Methodological Index for Non-randomized Studies, *NR* not reported.

The included studies consisted of 127 patients (130 knees) with a mean age of 32.4 ± 8.8 years. On average, men comprised 46.7% ± 22.3% of the included patients. The average follow-up was 5.5 ± 1.9 years. Five studies reported RTS rates with a mean age of 33.8 ± 11.3 years and men comprising 39.5% ± 14.4% of study samples [1,2,11,41,55]. Four studies used the lateral opening-wedge technique [1,2,41,42], 1 study used the wedgeless V-shaped technique [11], and 2 studies used a combination of both the lateral opening-wedge and medial closing-wedge technique [43,55]. No studies reported the use of navigation. Three studies reported RTW rates with a mean age of 36.4 ± 8.5 years and men comprising 48.4% ± 25.4% of study samples [11,42,43]. Notably, 1 of the studies included in this analysis reported both RTS and RTW rates [11]. Three studies were included despite being derived from a single-patient database due to mutually exclusive patient eligibility criteria and reported outcomes [1,41,42]. Specifically, only 1 of these studies reported RTW rates [42], whereas the other 2 studies reported RTS rates for patient populations either undergoing isolated lateral opening-wedge DFVO or DFVO with lateral meniscal allograft transplant, 2 mutually exclusive procedures [1,41].

Only 2 studies reported explicit OA thresholds as an indication for DFVO [1,11]. Three studies stated that “symptomatic osteoarthritis” was an indication for DFVO [11,42,55], and 2 studies reported symptoms specific for concurrent meniscal allograft transplantation [41,42]. Two studies reported minimum thresholds for valgus malalignment requiring DFVO [41,42]. Study-specific eligibility criteria can be found in Supplemental Table 2.

Six studies reported details regarding rehabilitation protocols [1,2,11,41,42,55]. All of these studies reported a 6-week period of either nonweightbearing [2,11,41,42] or limited weightbearing [1,55]. The use of a hinged knee brace was reported in the same 6 studies [1,2,11,41,42,55]. Full weightbearing as tolerated was initiated between 6 and 8 weeks postoperatively [1,2,11,41,42,55]. Formal physiotherapy initiation was reported by 2 studies [2,41], with 1 of these studies explicitly reporting a standardized physiotherapy start date of 10 days postoperatively [41].

Five studies reported RTS rates (Supplemental Table 3) [1,2,11,41,55]. The mean RTS rate irrespective of returning to preoperative activity levels was 87.2% ± 10.7% with an average RTS time of 12.3 ± 3.4 months [1,2,11,41,55]. Four studies reported details regarding activity intensity relative to preoperative levels with a mean RTS rate to preoperative levels or better of 65.4% ± 26.8% [1,2,11,41]. Three studies included a combination of collegiate, high school, and recreational athletes [1,41,55], 1 study included only elite level athletes at the collegiate level [2], and 1 study included patients participating in an unspecified level of sporting activity [11].

Of the 3 studies that reported RTW rates (Supplemental Table 3), 2 studies specified activity levels at work [42,43] and 1 study only provided details regarding work responsibility relative to preoperative levels [11]. The mean RTW rate irrespective of preoperative activity level was 81.8% ± 23.3%. The mean RTW rate to preoperative activity levels or better was 72.8% ± 18.1% (95% CI, 52.3%–93.3%). One study reported a mean RTW time of 6.0 ± 13.2 months [42]. Five studies reported preoperative and postoperative validated pain-related outcome scores [11,41,43,55] (Supplemental Table 4). All of these reported statistically significant improvements from preoperative to postoperative states (*P* < .05).

Four studies reported a mean preoperative valgus alignment of 9.1° ± 2.7° (95% CI, 6.5°–11.7°) [2,11,41,55]. Two studies reported a mean postoperative alignment of 0° ± 0° [11,55]. Study-specific details can be found in Supplemental Table 4.

All included studies reported rates of concomitant procedures and reoperations following the index DFVO surgery, with the exception of 1 study that only reported concomitant procedures [11]. The mean number of concomitant procedures per knee was 0.7 ± 0.6 (95% CI, 0.3–1.1) with details provided in Supplemental Table 5. The mean postoperative reoperation rate was 35.6% ± 18.8% (95% CI, 20.6%–50.7%) with details provided in Supplemental Table 6. The mean conversion to TKA rate was 4.0% ± 2.9% (95% CI, 1.6%–6.3%) at 4.9 ± 2.4 years postoperatively.

## Discussion

This study’s primary finding is that RTS and RTW rates following DFVO were similar, although moderately variable at 87.2% ± 10.7 and 81.8% ± 23.3%, respectively. The average time to RTS and RTW was also heterogenous. Despite the variability, DFVO is effective at reducing pain secondary to valgus malalignment of the knee. We also found a low rate of conversion to TKA of 4.0% ± 2.9% at approximately 5 years postoperatively.

This study was not without its limitations. First, all included studies were level IV noncomparative case series. Although the studies’ quality was moderate based on the MINORS quality assessment tool, they consistently lacked a prospective study design and prospective calculation of study size. Second, while we attempted to adjust for age when comparing RTS and RTW rates across DFVO and TKA, our review’s mean age remained significantly lower than that of the available TKA literature. Third, although 1 of the often-cited clinical benefits of DFVO is that it enables young patients to return to activity and work, this study found very limited literature explicitly reporting either RTW or RTS rates. Fourth, among studies that did report RTS and RTW rates, there were highly variable populations, outcomes, indications for surgery, and surgical techniques used. This rendered it challenging to ascertain which factors most affected postoperative outcomes. Therefore, not only should future studies evaluating the efficacy of DFVOs explicitly report RTS and RTW rates, a standardized reporting methodology should be adopted to strengthen the generalizability of the findings.

The DFVO for valgus malalignment of the knee in young, active patients has some advantages over TKA as it enables patients to return to high-impact activities while also delaying any long-term implant complications associated with TKA [50]. This review found that RTW and RTS rates following DFVO were relatively higher compared with reported rates following TKA, even when adjusting for age. A recent prospective study by Scott et al [49] demonstrated that in a working population with a mean age of 59 years, 40% of patients returned to work following TKA. This is considerably lower than our study’s post-DFVO RTW rate of 81.8%. Furthermore, a systematic review by Witjes et al [58] found that RTS rates following TKA in all comers (ie, not specifically valgus or young patients) ranged from 36% to 89%, which is appreciably lower relative to our review’s post-DFVO RTS rates ranging from 70.6% to 100%. In the 3 studies in Witjes et al [58] that analyzed RTS rates [4,22,27] in those less than 65 years [36], RTS rates ranged from 57% to 89%, lower than our study’s rates. Although Witjes et al did not specify if patients returned to preoperative activity levels, previous literature has demonstrated that following TKA, patients often return to decreased frequency [22] and intensity [5] of activity. Our study found a similar trend in the DFVO population with mean RTS rate to preoperative activity levels or better being 65.4% ± 26.8% compared with an absolute mean RTS rate of 87.2% ± 10.7%. Overall, it seems likely that both RTS and RTW rates are better for patients undergoing DFVO compared with TKA, although patients undergoing DFVO are generally younger than those undergoing TKA, which is likely a confounding factor.

With the exception of 1 study that reported RTW rates in young active military personnel [43], we found RTW rates that were comparable with the entire TKA population [16]. Two included studies reported RTW rates to preoperative activity levels or better of 78.2% and 88.5% [11,42], which is comparable with reported RTW rates in the TKA population of 81.5% to 89% [16,31,48]. We did find that mean RTW rate to preoperative activity levels was lower than the absolute RTW rate (72.8% ± 18.1% vs 81.8% ± 23.3%). Therefore, while DFVO is an effective intervention at allowing patients to RTW while also delaying TKA, it may not reliably return patients back to rigorous professions. Similar to HTO for varus knee deformity [21], it is not uncommon for patients to return to a decreased level of activity following DFVO [1,41,42]. Considering the promising RTW rates following DFVO, this intervention can effectively delay the need for TKA in younger patients with valgus malalignment of the knee while also allowing them to stay active and employed in the interim.

A notable trend in both of our review’s RTS and RTW rates is that not all patients were able to return to their preoperative activity levels. Based on this review’s findings, approximately 25% and 11% of patients returning to sport or work, respectively, may not be able to return to their preoperative intensity levels. This is not unique to the DFVO population. A systematic review by Ekhtiari et al [13] examining RTS and RTW rates following HTO for varus malalignment of the knee suggests that 10% and 22% of patients returning to sport or work will not be able to return to their preoperative activity levels, respectively. Limitations to returning to preoperative activity levels, or better, persist in the TKA population with 3% [20] and 9% to 16% [28,31,49] of patients returning to sport and work, respectively, being unable to do so at their preoperative intensity levels. The mean age of patients followed in the studies evaluating RTS and RTW rates in the TKA population was considerably higher than the mean age of patients in our included studies, which limits the comparability of these findings [20,28,31,49]. Patients undergoing DFVO should be counseled regarding the probability of being unable to return to their preoperative activity levels. Future studies are warranted to further delineate the impact of preoperative activity levels and patient characteristics on RTS and RTW rates following DFVO.

This study found a total reoperation rate of 34.6% and a lesser reoperation rate of 25.7% when excluding hardware removal procedures (an often-planned reoperation following DFVO). A previous systematic review by Wylie et al [60] found pooled reoperation rates of 35% and 44% following medial closing-wedge and lateral opening-wedge DFVOs, respectively. While largely comparable with previously reported rates in the literature, we did find that our included retrospective review by Rensing et al [43] had a uniquely high reoperation rate of 54.5%. Considering this patient cohort comprised active military personnel, it is possible that increased levels of postoperative activity may be associated with increased reoperation rates. Furthermore, this systematic review found that the rate of conversion to TKA following DFVO was 4.0% ± 2.9% within follow-up periods ranging from 3 to 7.5 years. Although comparable with the literature, it has been demonstrated that DFVO survival rates are inversely proportional to time since the index surgery [7]. Therefore, future DFVO studies should implement long-term follow-up periods to elucidate the effect various activity levels have on reoperation and conversion rates.

In conclusion, our systematic review of low-level studies found DFVO to be a safe and effective procedure for the management of genu valgum in young, active populations. The rates of RTW and RTS have been demonstrated to be slightly favorable to those of TKA, in addition to maintaining native joint mechanics and preserving bone stock. The DFVO for genu valgum is also effective at reducing associated pain. Future studies should implement strong research methodology including a prospective study design, explicit patient selection criteria, and a thorough definition of RTS and RTW to better ascertain the efficacy of DFVO on activity levels in young active patients. Future studies should also consider the effect of novel surgical techniques such as navigation assistance on radiographic and clinical outcomes.

## Supplemental Material

sj-docx-1-hss-10.1177_15563316211051295 – Supplemental material for Return to Sport and Work Following Distal Femoral Varus Osteotomy: A Systematic Review Click here for additional data file. Supplemental material, sj-docx-1-hss-10.1177_15563316211051295 for Return to Sport and Work Following Distal Femoral Varus Osteotomy: A Systematic Review by Hassaan Abdel Khalik, Darius L. Lameire, Luc Rubinger, Seper Ekhtiari, Vickas Khanna and Olufemi R. Ayeni in HSS Journal®: The Musculoskeletal Journal of Hospital for Special Surgery

sj-docx-2-hss-10.1177_15563316211051295 – Supplemental material for Return to Sport and Work Following Distal Femoral Varus Osteotomy: A Systematic Review Click here for additional data file. Supplemental material, sj-docx-2-hss-10.1177_15563316211051295 for Return to Sport and Work Following Distal Femoral Varus Osteotomy: A Systematic Review by Hassaan Abdel Khalik, Darius L. Lameire, Luc Rubinger, Seper Ekhtiari, Vickas Khanna and Olufemi R. Ayeni in HSS Journal®: The Musculoskeletal Journal of Hospital for Special Surgery

sj-docx-3-hss-10.1177_15563316211051295 – Supplemental material for Return to Sport and Work Following Distal Femoral Varus Osteotomy: A Systematic Review Click here for additional data file. Supplemental material, sj-docx-3-hss-10.1177_15563316211051295 for Return to Sport and Work Following Distal Femoral Varus Osteotomy: A Systematic Review by Hassaan Abdel Khalik, Darius L. Lameire, Luc Rubinger, Seper Ekhtiari, Vickas Khanna and Olufemi R. Ayeni in HSS Journal®: The Musculoskeletal Journal of Hospital for Special Surgery

sj-docx-4-hss-10.1177_15563316211051295 – Supplemental material for Return to Sport and Work Following Distal Femoral Varus Osteotomy: A Systematic Review Click here for additional data file. Supplemental material, sj-docx-4-hss-10.1177_15563316211051295 for Return to Sport and Work Following Distal Femoral Varus Osteotomy: A Systematic Review by Hassaan Abdel Khalik, Darius L. Lameire, Luc Rubinger, Seper Ekhtiari, Vickas Khanna and Olufemi R. Ayeni in HSS Journal®: The Musculoskeletal Journal of Hospital for Special Surgery

sj-docx-5-hss-10.1177_15563316211051295 – Supplemental material for Return to Sport and Work Following Distal Femoral Varus Osteotomy: A Systematic Review Click here for additional data file. Supplemental material, sj-docx-5-hss-10.1177_15563316211051295 for Return to Sport and Work Following Distal Femoral Varus Osteotomy: A Systematic Review by Hassaan Abdel Khalik, Darius L. Lameire, Luc Rubinger, Seper Ekhtiari, Vickas Khanna and Olufemi R. Ayeni in HSS Journal®: The Musculoskeletal Journal of Hospital for Special Surgery

## Appendix

**Appendix.** Search strings.

MEDLINE	EMBASE	Web of Science
1. Genu Valgum/su [Surgery] 2. distal femur osteotom*.mp. 3. distal femoral osteotom*.mp. 4. distal femur varus osteotom*.mp. 5. distal femoral varus osteotom*.mp. 6. (distal femur adj10 osteotom*).mp. 7. (distal femoral adj10 osteotom*).mp. 8. (osteotom* adj10 (valgus adj5 knee*)).mp. 9. 1 or 2 or 3 or 4 or 5 or 6 or 7 or 8 10. limit 9 to humans 11. limit 10 to English language	1. distal femur osteotom*.mp. 2. distal femoral osteotom*.mp. 3. distal femur varus osteotom*.mp. 4. distal femoral varus osteotom*.mp. 5. (distal femur adj10 osteotom*).mp. 6. (distal femoral adj10 osteotom*).mp. 7. valgus knee/su [Surgery] 8. (osteotom* adj10 (valgus adj5 knee*)).mp. 9. 1 or 2 or 3 or 4 or 5 or 6 or 7 or 8 10. limit 9 to human 11. limit 10 to English language	TS=(distal femoral osteotom*) OR TS=(distal femur osteotom*) OR TS=(distal femoral NEAR/10 osteotom*) OR TS=(distal femur NEAR/10 osteotom*) OR TS=(distal femur varus osteotom*) OR TS=(distal femoral varus osteotom*) OR TS=(osteotom* NEAR/10 (valgus NEAR/5 knee*))

*MEDLINE* Medical Literature Analysis and Retrieval System Online, *EMBASE* Excerpta Medica Database.
