# Supplementary material for: Return to Sport and Work Following Distal Femoral Varus Osteotomy: A Systematic Review
Source: HSS J. 2021 Oct 27;18(2):297–306. doi: 10.1177/15563316211051295 (PMC9096991; doi:10.1177/15563316211051295)
Supplement: sj-docx-1-hss-10.1177_15563316211051295 – Supplemental material for Return to Sport and Work Following Distal Femoral Varus Osteotomy: A Systematic Review [file sj-docx-1-hss-10.1177_15563316211051295.docx]

| **Table 2** Indications and Study-Specific Eligibility Criteria | | | | | | | |
| --- | --- | --- | --- | --- | --- | --- | --- |
|  |  | **Indications** | | |  | **Additional Indications/Eligibility Criteria** | |
| **Study** | **RTS or RTW Study?** | **Osteoarthritis** | **Symptoms** | **Valgus Malalignment** |  | **Inclusion Criteria** | **Exclusion Criteria** |
| Agarwalla (2020) | RTS | Isolated lateral compartment OA (Kellgren-Lawrence ≥ 1)  Kellgren-Lawrence III & IV, n(%): 8 (41.7) | None Stated | None stated |  | 1. Age <65 2. Isolated lateral compartment OA 3. Underwent isolated opening wedge DFVO 4. Minimum 2-year follow-up 5. Prior ipsilateral meniscectomy or ACLR included 6. Prior MAT, OAG, OATS, or ACI that were not part of a staged procedure were included | 1. 18 years or younger at the time of surgery  2. Bilateral DFVO within 3 years of each other. 3. Concomitant OAG, MAT, ACI |
| Baron (2020) | RTS | None stated | None stated | None stated |  | 1. High-level collegiate athletes undergoing DFVO | None stated |
| de Carvalho (2014) | RTS & RTW | Kellgren-Lawrence < 3 | Symptomatic OA with no further definition provided | None stated |  | None stated | 1. Grade III obesity (body mass index > 35 kg/m^2^) 2. Grade III or IV chondral lesions in the medial compartment 3. Symptomatic osteoarthritis of the patellofemoral joint 4. Movement arch < 90°  5. Local or systemic infections 6. Inflammatory arthropathies 7. Kellgren/Lawrence ≥ 5 III |
| Puzzitiello (2020)-a | RTS | None stated | *Specifically for MAT:* 1. Presence of knee pain unresponsive to conservative management for 6 months or longer 2. Knee pain that limits appropriate functional activity | ≥ 5° |  | 1. Patients who received simultaneous MAT and lateral opening wedge DFVO  *For MAT:* 2. Functionally meniscectomized in the lateral compartment (>50% absence) 3. Skeletal maturity 4. Minimal to absent degenerative changes in surrounding articular cartilage (Outerbride grade II or less) | 1. Concomitant procedures other than cartilage restoration procedures for focal full-thickness cartilage defects of the lateral femoral condyle, as well as bipolar defects |
| Puzzitiello (2020)-b | RTW | None stated | Symptomatic OA with no further definition provided  Specifically for MAT: 1. Presence of knee pain unresponsive to conservative management for 6 months or longer 2. Knee pain that limits appropriate functional activity | ≥ 5° |  | 1. Employed within 3 years prior to DFVO operation 2. Minimum of 2 year follow-up  *For MAT:* 3. Functionally meniscectomized in the lateral compartment (>50% absence) 4. Skeletal maturity 5. Minimal to absent degenerative changes in surrounding articular cartilage (Outerbridge grade II or less) | 1. Revision DFVOs 2. Bilateral DFVOs within 3 years of each other |
| Rensing (2019) | RTW | None stated  45.5% of patients had mild to severe radiographic evidence of osteoarthritis | None stated | None stated |  | 1. Active duty service member undergoing DFVO for primary valgus deformity | 1. Non-active duty military personnel 2. Correction of post-traumatic malunions or rotational deformity |
| Voleti (2019) | RTS | Lateral compartment OA on imaging (no formal criteria reported) | Symptomatic lateral compartment overload with no improvement following nonoperative management. No specifics provided. | None stated |  | 1. Concomitant procedures were permitted  2. Participated in competitive sports prior to DFVO defined as 4 or more days of activity per week | None stated |
| RTS, return to sport; RTW, return to work; DFVO, distal femoral varus osteotomy; MAT, meniscal allograft transplant; OAG, osteochondral allograft; OATS, osteochondral autograft transfer system; ACI, autologous chondrocyte implantation; OA, osteoarthritis | | | | | | | |
