# Supplementary material for: Return to Sport and Work Following Distal Femoral Varus Osteotomy: A Systematic Review
Source: HSS J. 2021 Oct 27;18(2):297–306. doi: 10.1177/15563316211051295 (PMC9096991; doi:10.1177/15563316211051295)
Supplement: sj-docx-2-hss-10.1177_15563316211051295 – Supplemental material for Return to Sport and Work Following Distal Femoral Varus Osteotomy: A Systematic Review [file sj-docx-2-hss-10.1177_15563316211051295.docx]

| **Table 3** Procedures and Return to Work (RTW) and Return to Sport (RTS) Outcomes | | | | | | |
| --- | --- | --- | --- | --- | --- | --- |
|  |  |  | **Outcomes** | | | |
| **Study** | **Procedure (%)** | **Definition** | **Pre-Operative** | **Post-Operative** | **Mean Time to RTS/RTW, months** | **RTS/RTW Rate**  **(Pre-Op Activity Level or Better), %** |
| ***Return to Sport*** | | | | | | |
| Agarwalla (2020) | Lateral open wedge: 100 | Return to sport at pre-injury level of sport or higher | Collegiate: 3 (17.6%) Competitive: 2 (11.8%) Recreational: 12 (70.6%) | Collegiate: 0 (0.0%) Competitive: 2 (11.8%) Recreational: 10 (58.8%) | 9.5 ± 3.3 (range, 3-12) | 70.6 (35.3) |
| Baron (2020) | Lateral open wedge: 100 | Return to contact collegiate sports | 1. 17M high school basketball player 2. 19F NCAA Division I collegiate basketball player 3. 21F NCAA Division I collegiate basketball player | 1. Returned to sport at 10 months, obtained Division I scholarship offer 2. Returned to Division I collegiate basketball sport at 9 months 3. Returned to Division I collegiate basketball at 6 months | 8.3 ± 2.1 (range, 6-10) | 100 (100) |
| de Carvalho (2014) | Wedgeless V-shaped: 100 | Defined as per patient | 15 patients reported routine physical activity (hydrogymnastics, hydrotherapy, swimming, soccer, volleyball) | 14 patients resumed their routine physical activity (all those apart from volleyball) | NR | 93.3 (93.3) |
| Puzzitiello (2020)-a | Lateral open wedge: 100 | Return to sport at pre-injury level of sport or higher | Collegiate: 4 (23.5%) Competitive: 3 (17.6%) Recreational: 8 (47.1%) | Collegiate: 1 (5.9%) Competitive: 3 (17.6%) Recreational: 10 (58.8) | 16.9 (range, 6-36) | 82.4 (46.7) |
| Voleti (2019) | Lateral open wedge: 53.8 Medial closing wedge: 46.2 | Return to sport was defined as returning to athletic activity at least 4 days per week | Rec soccer: 3 Softball: 2 Running: 2 HS basketball: 1 HS hockey: 1 Rec volleyball: 1 HS soccer: 1 Football: 1 Rower: 1 | Rec soccer: 4 Softball: 2 Running: 2 Rec basketball: 1 Rec football: 1 Rec hockey: 1 Rec volleyball: 1 Rower: 1 | 11 (range, 9-13) | 100 (NR) |
| **Weighted mean ± SD** | | |  |  | **12.3 ± 3.4** | **87.2 ± 10.7**  **(65.4 ± 26.8)** |
| ***Return to Work*** | | | | | | |
| de Carvalho (2014) | Wedgeless V-shaped: 100 | Normal work duties at preoperative functional level without any limitations or decline in performance | 26 patients | 23 patients | NR | 88.5 (88.5) |
| Puzzitiello (2020)-b | Lateral open wedge: 100 | Work intensity stratified as sedentary/light-, moderate- and heavy-intensity | Sedentary: 4 (12.5%) Light: 4 (12.5%) Moderate: 16 (50%) Heavy: 8 (25%) | Sedentary: 4 (12.5%) Light: 7 (21.9%) Moderate: 17 (53.1%)  Heavy: 4 (12.5%) | Sedentary: 2.7 months Light: 2.9 months Moderate: 3.1 months Heavy: 13.8 months  **Mean:** **6.0 ± 13.2** | 100 (78.2) |
| Rensing (2019) | Lateral open wedge: 90.9 Medial closing wedge: 9.1 | Continued military service 2 years following DFVO | 22 active service members | 11 active service members @ 2 years | NR | 42.1 (42.1) |
| **Weighted mean ± SD** | | |  |  | **N/A** | **81.8 ± 23.3**  **(72.8 ± 18.1)** |
| NR, not reported | | |  |  |  |  |
