# Supplementary material for: Return to Sport and Work Following Distal Femoral Varus Osteotomy: A Systematic Review
Source: HSS J. 2021 Oct 27;18(2):297–306. doi: 10.1177/15563316211051295 (PMC9096991; doi:10.1177/15563316211051295)
Supplement: sj-docx-3-hss-10.1177_15563316211051295 – Supplemental material for Return to Sport and Work Following Distal Femoral Varus Osteotomy: A Systematic Review [file sj-docx-3-hss-10.1177_15563316211051295.docx]

| **Table 4** Secondary Outcomes | | | | | | | |
| --- | --- | --- | --- | --- | --- | --- | --- |
|  |  | **Radiographic Alignment^†^**  *mean ± SD (range)* | | **Pain,**  *mean ± SD (range)* | | |  |
| **Study (Year)** | **RTS or RTW** | **Pre-Operative** | **Post-Operative** | **Outcome Measure** | **Pre-Operative** | **Post-Operative** | **Reoperation Rate (%)** |
| Agarwalla (2020) | RTS | NR | NR | SANE (/100) | NR | 56.2 ± 18.7 (20-85) | 41.2% |
| Baron (2020) | RTS | Case 1: 8° valgus Case 2: Not reported Case 3: 8° valgus | Case 2: neutral alignment | NR | NR | NR | 33.3% |
| de Carvalho (2014) | RTS & RTW | 12° ± 2° (8°-15°) | °0 ± 1° (-1°-1°)* | Lysholm (/100) | 53.1 ± 16.2 (24-95) | 77.3 ± 16.7 (29-100)* | NR |
| Puzzitiello (2020)-a | RTS | 6.3° ± 1.3° (5°-8.5°) | NR | SANE (/100)  VAS Pain Score (/10) | 42.4 ± 21.6 (17-65)  6.7 ± 1.9 (4-10) | 61.5 ± 25.3 (30-95)  2.6 ± 2.3 (0-7)* | 11.8% |
| Puzzitiello (2020)-b | RTW | NR | NR | VAS Pain Score (/10) | 6.1 | 3.2* | 40.6% |
| Rensing (2019) | RTW | Mean Zone: 1.9 | Mean Zone: 1 | VAS Pain Score (/10) | 4.0 ± 2.3 (0-7) | 1.9 ± 1.8 (0-6)* | 54.5% |
| Voleti (2019) | RTS | 7° (5°-13°) | 0° (0°-2° varus)* | IKDC Score (/100) | 52.8 ± 10.9 (32-69) | 89.2 ± 5.7 (78-96)* | 7.7% |
| RTS, return to sport; RTW, return to work; NR, not reported; *p<0.05; ^†^valgus alignment unless otherwise stated; SANE, single assessment numeric evaluation; VAS, visual analogue scale’ IKDC, International Knee Documentation Committee | | | | | | | |
