# Supplementary material for: Return to Sport and Work Following Distal Femoral Varus Osteotomy: A Systematic Review
Source: HSS J. 2021 Oct 27;18(2):297–306. doi: 10.1177/15563316211051295 (PMC9096991; doi:10.1177/15563316211051295)
Supplement: sj-docx-4-hss-10.1177_15563316211051295 – Supplemental material for Return to Sport and Work Following Distal Femoral Varus Osteotomy: A Systematic Review [file sj-docx-4-hss-10.1177_15563316211051295.docx]

| **Table 5** Concomitant Procedures | | | | | | | | | | | | | |
| --- | --- | --- | --- | --- | --- | --- | --- | --- | --- | --- | --- | --- | --- |
| **Author (Year)** | **MAT** | **LFC Osteochondral Allograft** | **Unspecified Cartilage Grafting** | **Meniscectomy** | **Chondroplasty** | **Lysis of Adhesions, Debridement, or Loose Body Removal** | **LFC or Tibial Microfracture** | **Revision ACLR** | **Autologous Chondrocyte Implantation** | **MPFL Reconstruction** | **TTO** | **Total Concomitant Procedures** |  |
| Agarwalla (2020) | - | - | - | - | - | - | - | - | - | - | - | **-** |  |
| Baron (2020) | - | - | - | 1 | 1 | 1 | 1 | - | - | - | - | **4** |  |
| de Carvalho (2014) | - | - | - | 6 | 6 | - | - | - | - | - | - | **12** |  |
| Puzzitiello (2020)-a | 17 | 14 | - | - | - | - | 2 | - | 1 | - | - | **34** |  |
| Puzzitiello (2020)-b | 11 | - | 12 | - | - | - | - | - | - | - | - | **23** |  |
| Rensing (2019) | 1 | - | - | - | 2 | 3 | - | 1 | - | 1 | 1 | **9** |  |
| Voleti (2019) | 1 | 6 | - | 2 | - | 2 | - | 1 | - | - | - | **12** |  |
| **Total** | **30** | **20** | **12** | **9** | **9** | **6** | **3** | **2** | **1** | **1** | **1** | **94** |  |
| MAT, meniscal allograft transplantation; LFC, lateral femoral condyle; ACLR, anterior cruciate ligament reconstruction; MPFL, medial patellofemoral ligament | | | | | | | | | | | | | |
