# Supplementary material for: Return to Sport and Work Following Distal Femoral Varus Osteotomy: A Systematic Review
Source: HSS J. 2021 Oct 27;18(2):297–306. doi: 10.1177/15563316211051295 (PMC9096991; doi:10.1177/15563316211051295)
Supplement: sj-docx-5-hss-10.1177_15563316211051295 – Supplemental material for Return to Sport and Work Following Distal Femoral Varus Osteotomy: A Systematic Review [file sj-docx-5-hss-10.1177_15563316211051295.docx]

| **Table 6** Postoperative Reoperations |  |
| --- | --- |
| **Reoperation^†^** | **Mean Rate, % (SD)** |
| Hardware removal | 9.9 (8.2) |
| Hardware removal with additional ligamentous reconstruction | 3.0 (4.4) |
| ACLR ± meniscal transplantation ± additional ligamentous reconstruction | 2.0 (4.1) |
| TKA | 4.0 (2.9) |
| Bone grafting procedure | 2.0 (4.1) |
| Revision DFVO | 2.0 (2.1) |
| Manipulation under anesthesia | 5.0 (7.3) |
| MAT, OATS or combination | 3.0 (4.2) |
| Meniscal debridement/meniscectomy | 4.0 (8.8) |
| MPFL reconstruction with patellar chondroplasty | 1.0 (2.1) |
|  |  |
| **Total Rates** |  |
| Reoperation rate including hardware removal | 35.6 (18.8) |
| Reoperation rate excluding hardware removal | 25.7 (16.3) |
| ^†^Reoperations from most to least common; ACLR, anterior cruciate ligament reconstruction; TKA, total knee arthroplasty; DFVO, distal femoral varus osteotomy; MAT, meniscal allograft transplantation; MPFL, medial patellofemoral ligament | |
